# Exploring the Antiangiogenic Potential of Solomonamide A Bioactive Precursors: In Vitro and In Vivo Evidences of the Inhibitory Activity of Solo F-OH During Angiogenesis

**DOI:** 10.3390/md17040228

**Published:** 2019-04-15

**Authors:** Paloma Carrillo, Beatriz Martínez-Poveda, Iván Cheng-Sánchez, Jessica Guerra, Chiara Tobia, J. Manuel López-Romero, Francisco Sarabia, Miguel Ángel Medina, Ana R. Quesada

**Affiliations:** 1 Department of Molecular Biology and Biochemistry, Faculty of Sciences, University of Málaga, Andalucía Tech, 29071 Málaga, Spain; pcarrillo@uma.es (P.C.); medina@uma.es (M.Á.M.); quesada@uma.es (A.R.Q.); 2 IBIMA (Biomedical Research Institute of Málaga), 29071 Málaga, Spain; 3 Department of Organic Chemistry, Faculty of Sciences, University of Málaga, Andalucía Tech, 29071 Málaga, Spain; cheng@uma.es (I.C.-S.); jmromero@uma.es (J.M.L.-R.); frsarabia@uma.es (F.S.); 4 Department of Molecular and Translational Medicine, Experimental Oncology and Immunology, School of Medicine, University of Brescia, 25125 Brescia, Italy; j.guerra@unibs.it (J.G.); chiara.tobia@unibs.it (C.T.); 5 CIBER of Rare Diseases, Group U741 (CB06/07/0046), 29071 Málaga, Spain

**Keywords:** Solomonamide A, marine sponge, endothelial cells, angiogenesis, cancer

## Abstract

Marine sponges are a prolific source of bioactive compounds. In this work, the putative antiangiogenic potential of a series of synthetic precursors of Solomonamide A, a cyclic peptide isolated from a marine sponge, was evaluated. By means of an in vitro screening, based on the inhibitory activity of endothelial tube formation, the compound Solo F–OH was selected for a deeper characterization of its antiangiogenic potential. Our results indicate that Solo F–OH is able to inhibit some key steps of the angiogenic process, including the proliferation, migration, and invasion of endothelial cells, as well as diminish their capability to degrade the extracellular matrix proteins. The antiangiogenic potential of Solo F–OH was confirmed by means of two different in vivo models: the chorioallantoic membrane (CAM) and the zebrafish yolk membrane (ZFYM) assays. The reduction in ERK1/2 and Akt phosphorylation in endothelial cells treated with Solo F–OH denotes that this compound could target the upstream components that are common to both pathways. Taken together, our results show a new and interesting biological activity of Solo F–OH as an inhibitor of the persistent and deregulated angiogenesis that characterizes cancer and other pathologies.

## 1. Introduction

Marine sponges are considered as a rich source of new potential bioactive compounds. In living sponges, these compounds are mainly secondary metabolites, whose natural functions include defense against predators and competition with other sessile species. The increasing interest for sponge-isolated compounds in biomedical and pharmaceutical research derives from the potential clinical applications exhibited by them, according to their antitumoral, anti-inflammatory, antiangiogenic, and/or antimicrobial properties reported [1,2,3,4,5,6,7].

Several compounds isolated from the marine sponge *Theonella swinhoei* have been studied because of their interesting bioactive properties. Such is the case of the potent anti-inflammatory octacyclopeptides perthamides [8,9] and swinholides, with antifungal effect [10] and antiproliferative activity against a number of tumor cells by the disruption of actin cytoskeleton [11]. Together with them, solomonamide A (**1**) and B (**2**) are two bioactive compounds that have been recently isolated from *T. swinhoei* and described as exerting a dose-dependent anti-inflammatory activity in vivo [12,13].

Structurally, solomonamides are cyclic peptides that present in their structures an unusual peptide with non-proteinogenic amino acids. Thus, an extensive spectroscopic study allowed their structural determination, revealing the presence of three conventional amino acids (d-Ala, Gly, and l-Ser) and an unprecedented 4-amino(2-amino-4-hydroxyphenyl)-3,5-dihydroxy-2-ethyl-6- oxohexanoic acid (ADMOA) and its corresponding 5-deoxy derivative (AHMOA) for solomonamides A and B, respectively. These peptide structures provide protection against peptidases and constitute a scaffold for new compounds with better biological activities [14,15].

**Figure 1:**
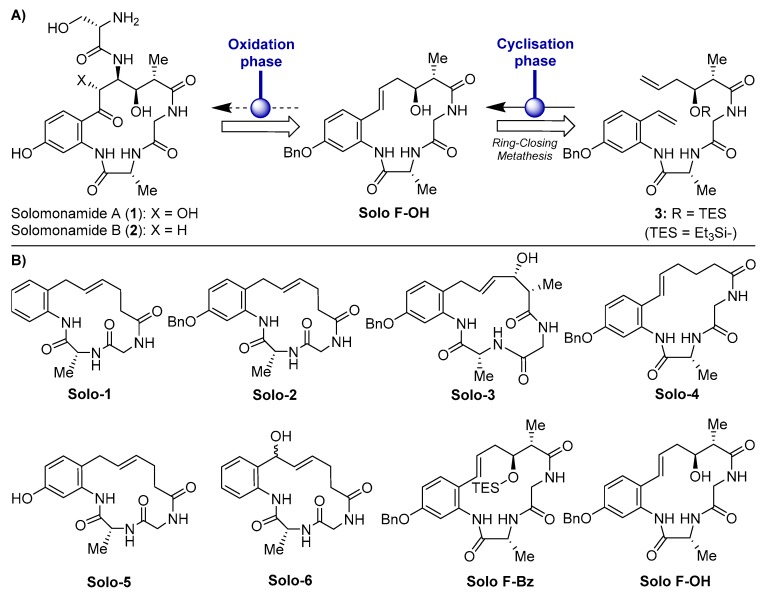
(**A**) Molecular structures of solomonamides A and B and synthetic strategy via ring-closing metathesis (RCM). (**B**) Chemical structures of the solomonamide precursors synthesized in Cheng-Sánchez et al. [17] and screened in this study for antiangiogenic activity.

The development of marine compounds may be hampered by a “problem of supply” [16]. As in the case of other bioactive compounds isolated from marine sponges, the limitation for a deeper study of the properties of solomonamides derives from the difficulty of accessing enough biomass for its purification. Indeed, the particular geographical niche of *T. Swinhoei*, which was collected at the Solomon Islands, South Pacific, worsens this limitation. In order to easily obtain solomonamides, different strategies were developed to chemically synthesize them, and very recently, the total syntheses of solomonamides A and B was reported by Reddy et al. [13]. Previously, in an effort to reach the complete synthesis of the solomonamides, a new synthetic strategy directed toward these natural products was designed by our group [17]. The synthetic strategy developed in that work was based on a ring-closing metathesis (RCM) to construct the macrocyclic core via acyclic precursor **3**, followed by a subsequent oxidation phase to install all the functional groups contained in the solomonamides (Figure 1A). These synthetic studies gave us the opportunity to generate a set of analogues (Figure 1B), which were evaluated for their bioactivity in vitro by the measurement of their cytotoxicity profile against a panel of different cell lines, including endothelial and tumor cells [17].

Angiogenesis involves the generation of new capillaries by the sprouting of pre-existing vessels. Although in a healthy situation this process is tightly regulated by a balance of stimulators and inhibitors, being restricted to specific situations such as embryonic development, endometrial regulation, reproductive cycle, and wound repair, a persistent and deregulated angiogenesis is related to the course of many pathologies [18,19] and is considered one of the hallmarks of cancer [20]. In consequence, the pharmacological regulation of angiogenesis emerges as an attractive strategy for the treatment of cancer and other angiogenesis-dependent diseases [21,22,23]. Attending to this evidence, the active search for new compounds that are able to modulate angiogenesis is a crucial research strategy in order to discover potential antiangiogenic drugs with possible clinical applications. Our group is actively involved in the identification and characterization of new natural bioactive compounds and synthetic derivatives with multitarget antiangiogenic effects [24,25,26,27,28]. 

In the present study, we analyzed the antiangiogenic potential of a series of synthetic solomonamide intermediates (Figure 1B). As a result of a primary screening in vitro, one of those compounds (Solo F–OH) was selected and subjected to a more in-depth assessment of its antiangiogenic activity both in vitro and in vivo. The results presented here are the first experimental evidence showing the antiangiogenic potential of the synthetic precursors of the solomonamides.

## 2. Results

### 2.1. Effects of Solomonamide A Analogues in Tubular-Like Structures’ Formation of Endothelial Cells

**Figure 2:**
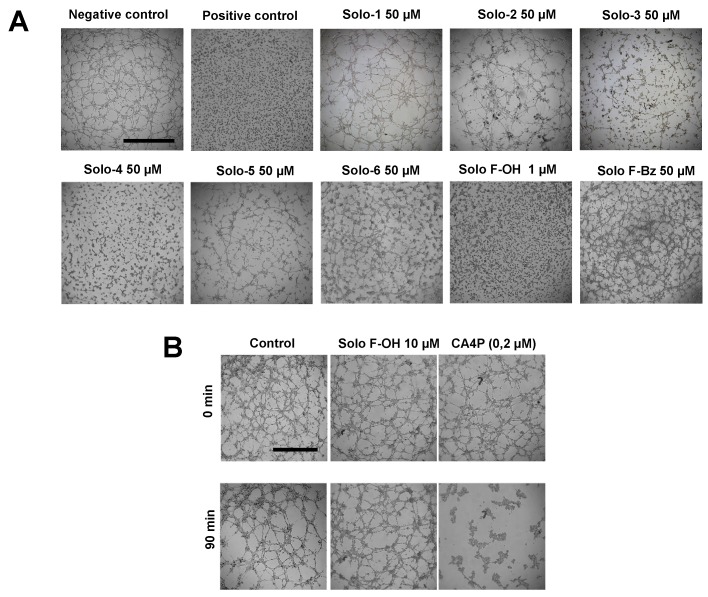
Solo F–OH exhibits a strong inhibitory effect on tubulogenesis in vitro, without affecting already formed structures. (**A**) Effect of solomonamide precursors on endothelial tubular-like structures formation on Matrigel. BAEC were seeded on Matrigel in presence of the compounds, and structures formation was evaluated after 5 h. Vehicle (DMSO) was added to the negative control; staurosporine (2 µM) was used as positive inhibition control (scale bar = 1000 µm). (**B**) Vascular disruption assay in vitro. Compounds were added to already formed BAEC tubular-like structures on Matrigel. Effects were evaluated after 90 min. Combretastatin A-4 phosphate (CA4P, 0.2 µM) was used as a positive control (scale bar = 1000 µm). Each experimental condition was conducted in duplicates, and three independent assays were performed in each case.

In [17], we previously reported the IC_50_ values (determined at 72 h of treatment) of the different solomonamide precursors in bovine aortic endothelial cells (BAEC) growth (Table 1). To in vitro evaluate the possible antiangiogenic activity of these series of synthetic solomonamide precursors, we performed a primary screening based on the analysis of their capability to inhibit the formation of endothelial tubular-like structures on Matrigel, using staurosporine 2 μM as a positive control. We tested all the intermediates in this assay establishing their minimum inhibitory concentration (MIC). For most of the tested compounds, MIC values were equal to or higher than 50 μM (doses higher than 50 μM were not considered for the study) (Table 1 and Figure 2A). Interestingly, in this primary screening, we identified the compound Solo F–OH, which exhibited an MIC value of 1 μM (Table 1 and Figure 2A), which is a concentration that is markedly low compared to the MIC determined for the rest of solomonamide analogues tested, and 18-fold lower than its IC_50_ in BAEC (Table 1). The potent activity of Solo F–OH to inhibit the formation of endothelial tubular-like structures on Matrigel prompted us to select this compound for the further analysis of its antiangiogenic potential.

### 2.2. Solo F–OH Does Not Produce Disruption of Endothelial Tubular-Like Structures Formed on Matrigel

Given the inhibitory effect of Solo F–OH in tubulogenesis in vitro, we studied the capability of the compound to target already formed vessels, checking its ability to disrupt the endothelial tubular-like structures already formed on Matrigel. We used combretastatin A-4 phosphate, which is a well-known vascular disruptor agent, as a positive control. The obtained results showed that solo F–OH is not able to disrupt the tubule-like structures already formed in Matrigel, even at a concentration that is 10-fold higher than the MIC value calculated in the tube formation assay (Figure 2B).

### 2.3. Solo F–OH Decreases the Migratory Potential of Endothelial Cells

**Figure 3:**
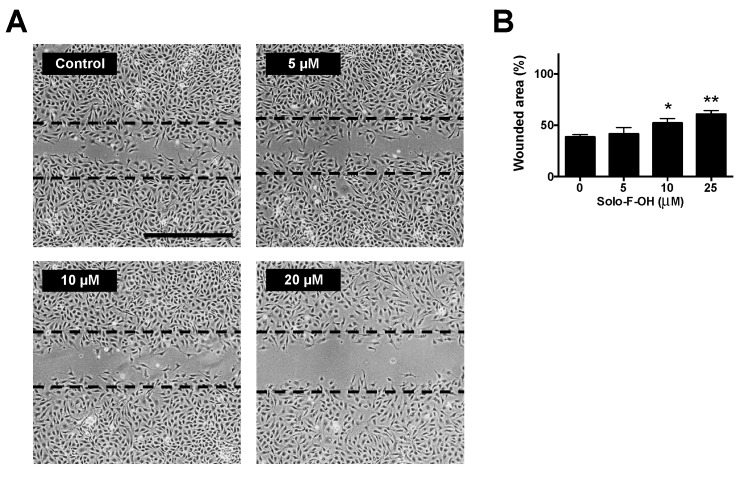
Solo F–OH decreases endothelial cell migration capability. (**A**) Representative photographs of wound-healing assay after 7 h of treatment with Solo F–OH. Vehicle (DMSO) was added to control condition. Discontinued lines point the free-cell area at time 0 h in each experimental condition. (Scale bar = 500 µm). (**B**) Quantification of the non-recovered area in the wound-healing assay after 7 h of treatment with Solo F–OH. Data are shown as percentages of the free-cell area at time 0 h, and are expressed as the mean ± SD of three independent experiments (* *p* < 0.05, ** *p* < 0.01).

During angiogenesis, migration is an indispensable step by which activated endothelial cells move toward the pro-angiogenic stimuli, making the formation of new vessels possible in the tissue. In order to evaluate the possible effect of the solomonamide precursors on endothelial cell migration in vitro, wound-healing assay was performed, and MIC values for migration inhibition were determined. As shown in Table 1, only the treatment with Solo F–OH decreased the migratory potential of endothelial cells, and no effect was observed with the other studied compounds at the concentrations tested after 7 h of treatment. Interestingly, the inhibitory effect of Solo F–OH on endothelial migration was dose-dependent (Figure 3), and the doses needed to inhibit migration were close or even lower to the IC_50_ value described for this compound in BAEC (Table 1).

### 2.4. Solo F–OH Inhibits the Invasive Capability of Endothelial Cells

**Figure 4:**
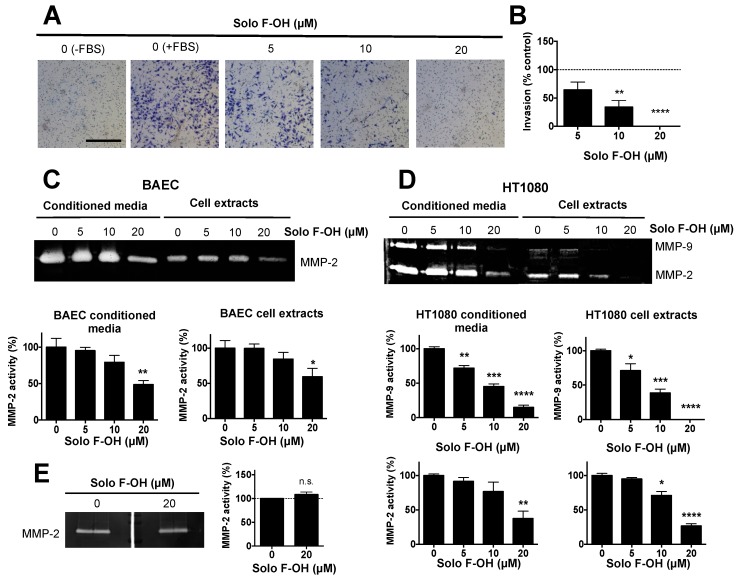
Solo F–OH inhibits endothelial cell invasion and extracellular matrix (ECM) degradation capability. (**A**) Representative photographs of invading endothelial cells through Matrigel-coated transwells after 16 h of treatment (Scale bar = 500 µm). (**B**) Quantification of the cell invasion assay. The number of invading cells stained in the control stimulated with fetal bovine serum (FBS) was considered as 100% of invasion. (**C**) Representative gelatin zymography and the quantification of MMP-2 presence in conditioned media and cell extracts of BAEC treated with Solo F–OH. (**D**) Representative gelatin zymographies and quantifications of MMP-9 and MMP-2 presence in conditioned media and cell extracts of HT1080 treated with Solo F–OH. (**E**) Representative gelatin zymography of untreated BAEC conditioned media, and the quantification of MMP-2 activity in the absence or presence of 20 μM of Solo F–OH added to the incubation buffer. For all the quantifications, data are the mean ± SD of at least three independent experiments (n.s., not significant; *p* < 0.05, ** *p* < 0.01, *** *p* < 0.001, **** *p* < 0.0001). MMPs: matrix metalloproteinases.

Additionally to the acquisition of migratory potential, during angiogenesis, activated endothelial cells must be able to degrade extracellular matrix components in order to allow the invasion through the tissue. The possible effect of Solo F–OH on the invasive capability of endothelial cells was studied by means of the invasion assay on Matrigel-coated transwells. As shown in Figure 4A,B, Solo F–OH was able to significantly inhibit the invasive potential of BAEC in a dose–response manner, reaching a 50% of inhibition at a concentration close to 5 μM. 

In addition, we studied the effect of Solo F–OH on the capability of endothelial cells to degrade the proteins of the extracellular matrix (ECM) by means of matrix metalloproteinases (MMPs). Gelatin zymography showed that the presence of MMP-2, the main MMP expressed in endothelial cells, was reduced both in cell extracts and in the conditioned media of BAEC treated with Solo F–OH at 20 μM (Figure 4C). These results supported the observed inhibitory effect of this compound on endothelial cell invasion. In order to study the cell specificity of the protease modulation by this compound, the effect of Solo F–OH on the proteolytic potential of HT1080 tumor cells was examined. While endothelial cells only express one gelatin degrading MMP, HT1080 cells express both gelatinases: MMP-2 and MMP-9. As shown in Figure 4D, both MMP activities were decreased when these cells were treated with Solo F–OH, suggesting that the inhibitory effect of Solo F–OH on the ECM-degrading potential is not specific for endothelial cells. In order to better understand the effect of Solo F–OH on ECM degradation capability, the in situ inhibition of MMP-2 activity was studied. Conditioned media of untreated BAEC were subjected to zymographic assays, and gelatinase activity was measured in the gel in the absence or presence of the compound added to the incubation buffer. Degradation bands were unaffected when gels were incubated in the presence of 20 μM of Solo F–OH (Figure 4E), indicating that this compound was not a direct inhibitor of MMP-2. 

### 2.5. Solo F–OH Inhibits Angiogenesis In Vivo

**Figure 5:**
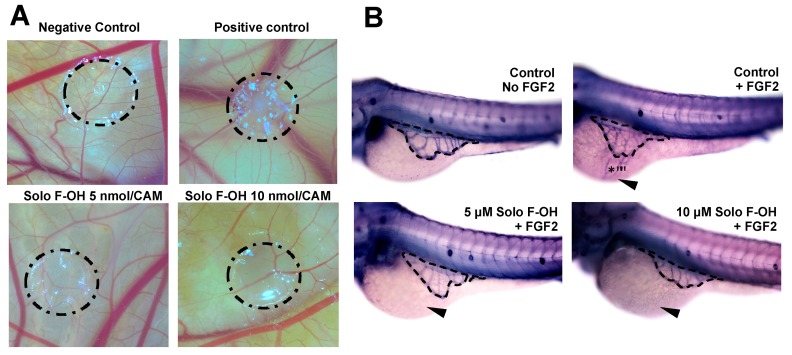
Solo F–OH shows a potent antiangiogenic effect in vivo. (**A**) Representative photographs of chorioallantoic membrane (CAM) assay testing Solo F–OH. Negative control condition containing vehicle (DMSO) and positive control condition containing aeroplysinin-1 (3 nmol/CAM) were used in the assay. Circles show the locations of the methyl cellulose discs. (**B**) Representative photographs of ZFYM assay. The response of subintestinal vessels of zebrafish embryos to fibroblast growth factor 2 (FGF-2)-induced angiogenesis in the presence of Solo F–OH was evaluated. Zebrafish embryos were stained for alkaline phosphatase activity. Dashes lines delimit a subintestinal vessels basket; arrowheads point to the FGF-2 injection site, and asterisks mark the angiogenic response to FGF-2.

The inhibitory activity of Solo F–OH on key steps of angiogenesis observed in vitro prompted us to study the in vivo effect of this compound by means of two different animal models. Firstly, Solo F–OH was tested in the chick CAM assay. In this assay, the presence of Solo F–OH into the methylcellulose discs clearly affected the normal development of vasculature in a dose-dependent manner, either inhibiting the ingrowth of new vessels in the area covered by the disc, or inducing rebounds of the peripheral ones (Figure 5A). As shown in Table 2, a dose-dependent effect of this compound on the neovascularization of the chorioallantoic membrane was observed, with a 50% of inhibition been reached at doses lower than 1 nmol/CAM. 

The in vivo Solo F–OH antiangiogenic activity was confirmed by using the zebrafish yolk membrane (ZFYM) assay. In this assay, we evaluated the in vivo effect of Solo F–OH in a background of fibroblast growth factor 2 (FGF-2)-induced angiogenesis. As shown in Figure 5B and summarized in Table 3, the treatment of embryos with different doses of Solo F–OH decreased the angiogenic response of subintestinal vessels (SIVs) toward the FGF-2 injection site. Indeed, the number of FGF-2-responsive embryos (both those exhibiting strong and mild response) was markedly reduced in presence of Solo F–OH. In parallel, the number of FGF-2-unresponsive embryos was increased with the treatments (Table 3).

### 2.6. Solo F–OH Interferes with the Activation of ERK1/2 and Akt Pathways

**Figure 6:**
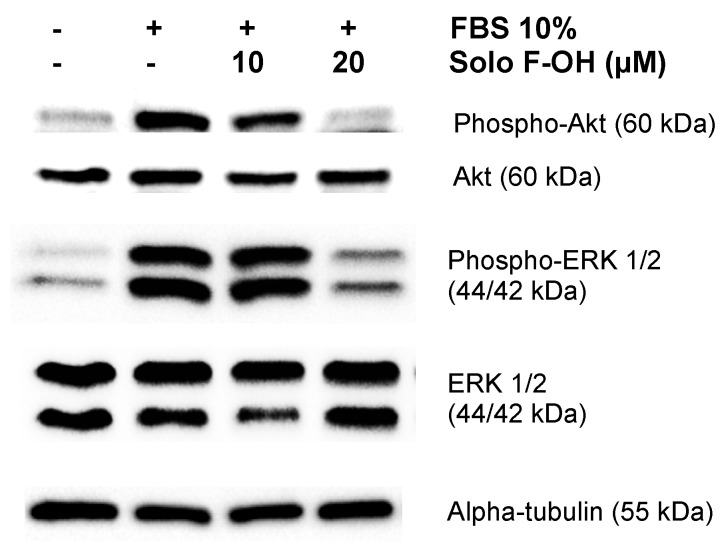
Solo F–OH inhibits AKT serine/threonine kinase (Akt) and extracellular signal-regulated kinase 1/2 (ERK1/2) phosphorylation in endothelial cells. Western blots of phosphorylated Akt, total Akt, phosphorylated ERK1/2, total ERK1/2, and alpha-tubulin in protein extracts from BAEC induced with serum in the absence or presence of Solo F–OH. Two independent experiments were performed with similar results.

Cellular processes related to angiogenesis are controlled by a complex network of signaling pathways in endothelial cells. Since PI3K/Akt and ERK–MAPK are two of the main signaling cascades implicated in the transduction of pro-angiogenic signals [29], we studied the effect of Solo F–OH in the activation of Akt and ERK1/2. Starved BAEC were induced with serum in the presence or absence of different doses of Solo F–OH, and the activation of Akt and ERK1/2 was measured by Western blot. As shown in Figure 6, the induction of BAEC with serum strongly increased Akt and ERK1/2 phosphorylation, indicating an effective activation of both pathways. In contrast, the phosphorylation of Akt and ERK1/2 in the presence of serum was substantially reduced when BAEC were treated with 20 μM of Solo F–OH (Figure 6).

### 2.7. Solo F–OH Does Not Inhibit the Tyrosine Kinase Activity of VEGFR2

Vascular endothelial growth factor receptor 2 (VEGFR2) plays a pivotal role in the deregulated connection of the “angiogenic switch”, which is characteristic of many angiogenesis-dependent diseases. Upon ligand binding, VEGFR2 undergoes autophosphorylation and becomes activated. Most of the clinically approved antiangiogenic drugs with low molecular weight inhibit the activation of VEGFR2, typically by inhibition of the receptor tyrosine kinase (TK) activity needed to initiate the VEGF signaling pathway [19,30]. In order to better characterize the molecular mechanism of action of Solo F–OH, we explored the effect of this compound on the TK enzymatic activity of the human recombinant VEGFR2 by means of a luminescent assay, which was designed to measure the remaining activity of the enzyme after 45 min of reaction at 30 °C in the absence or presence of tested compounds. Our results showed that the presence of Solo F–OH 20 μM did not significantly decrease the activity of the VEGFR2 TK activity in this assay (the remaining kinase activity relative to a vehicle control-DMSO was of 84.5 ± 3.3%, which was expressed as mean ± SD, results from four independent measures, *t*-test value versus control 0.16253). Sunitinib, a well-known inhibitor of VEGFR2 TK activity with a reported IC_50_ value of 0.08 μM in in vitro biochemical assays [31,32], was used as a positive control in these experiments, yielding a total inhibition of the kinase activity (0% of remaining activity) at a dose of 1 μM.

## 3. Discussion

Marine sponges produce a wide variety of singular metabolites that allow them to adapt and survive in their natural environment. Pharmacological interest in such metabolites mainly consists in the potential biomedical applications exhibited by many of them [1,2,3,4,5,6,7,33,34]. In this regard, several of these sponge-derived compounds or their synthetic analogues have been described as angiogenesis inhibitors [3,7,35,36].

The discovery of solomonamides in 2011 and the subsequent characterization of the potent anti-inflammatory activity of solomonamide A in vivo [12] opened the way to better decipher this and other possible bioactivities of the compounds. Despite this first report, the bioactivity of natural solomonamide A has not been further explored in our knowledge. The principal explanation to the lack of studies with such interesting compounds may be the shortage of natural resources needed for the molecule purification. Therefore, in the particular case of the solomonamides, the scarce availability of *T. swinhoei* clearly conditioned the further characterization of the compounds’ bioactivities. In the last years, different studies toward the synthesis of solomonamides have been reported, mainly by Reddy et al. [15,17,37,38,39,40,41]. In fact, very recently, the total syntheses of solomonamides A and B have been reported [13], which led to their stereochemical revision with respect to that initially proposed, and confirmed the anti-inflammatory activities reported for the natural products. 

Previously to the publication of the total synthesis, and due to the promising biological activity of solomonamide A, our group developed a chemical strategy aimed to explore the construction of the macrocycle core of the unprecedented cyclopeptide through an RCM reaction [17]. As a result of this study, different synthetic solomonamide precursors were obtained and further characterized in vitro for their cytotoxicity profile in a panel of endothelial and cancer cell lines. Their IC_50_ values showed that only one of those compounds (Solo F–OH in the present work) had a relevant cytotoxic activity in the totality of cell lines tested at the low micromolar range [17]. The promising results reported for Solo F–OH, together with our ongoing interest in the discovery and characterization of new antiangiogenic compounds [24,25,26,27,28], prompted us to evaluate the antiangiogenic potential of the complete series of solomonamide precursors in a primary screening in vitro for the inhibition of endothelial tubular-like structures formation on Matrigel. 

As for the growth inhibitory effect of this family of compounds [17], Solo F–OH displayed a highlighted inhibitory activity in endothelial tubular-like structure formation. This confirmed the unique bioactivity of this compound compared with the rest of the solomonamide precursors studied. In addition, the low MIC value exhibited in this assay by Solo F–OH (1 μM), which was around 20-fold lower than the dose required to inhibit BAE cell growth [17], points to a mechanism of action independent of its growth inhibitory activity in endothelial cells. Current approaches to target tumor vessels include antiangiogenic drugs, inhibiting the formation of new blood vessels following the activation of the endothelial cells, and tumor vascular disrupting drugs, in which the pre-existing vasculature is compromised and destroyed [42]. Our results show that Solo F–OH is not able to disrupt already formed endothelial tubular-like structures in vitro, discarding the possible vascular disrupting activity of this compound.

During the formation of the new vessel, endothelial cells activate their migratory and invasive potential, allowing the movement across the tissue toward the proangiogenic signal. Interestingly, Solo F–OH significantly reduced the migratory and invasive capabilities of BAEC in vitro at not-toxic doses. As a remark, the inhibition of endothelial cell migration showed by Solo F–OH in the wound-healing assay was determined after 7 h, which was a short time lapse at which cell proliferation is not relevant, suggesting that the antiproliferative activity of this compound is not playing a role in the inhibition of migration. Diminished endothelial cell invasion in the presence of Solo F–OH may be derived not only from the defective migratory capability observed after the treatment with the compound, but also its effect preventing endothelial ECM degradation by decreasing MMP-2, both in conditioned medium and cell extracts, in a dose-dependent manner. Our data showed that Solo F–OH is not a direct inhibitor of MMP-2 gelatinase activity, suggesting that the decrease in the degradative potential exerted by this compound could be due to an effect on the MMP-2 expression. MMP-2 is involved in angiogenesis regulation [43,44,45], and the decrease in secreted MMP-2 activity has been suggested to play a role in the inhibition of tubular-like structure formation and the reduction of endothelial cell migration by antiangiogenic natural compounds [46,47,48]. Such evidences could support the inhibitory effect observed for Solo F–OH in the formation of endothelial tubular-like structures and in cell migration.

In addition to the in vitro evidence of the inhibitory activity of Solo F–OH in different angiogenesis-related processes, the antiangiogenic potential of this compound was evaluated in vivo in two different models of angiogenesis: the CAM and the ZFYM assays. Firstly, the CAM assay allowed us to evaluate the effects of Solo F–OH in a context of physiological angiogenesis, which occurs during the development of the chick embryo. Our in vivo results derived from the CAM assay demonstrated that Solo F–OH was able to inhibit angiogenesis in a dose-dependent manner between 0.1–10 nmol/CAM, which is a very low concentration range in comparison with those reported for other known antiangiogenic compounds [49,50,51,52,53]. Secondly, the ZFYM assay allowed us to evaluate the ability of Solo F–OH to inhibit the angiogenesis induced by the exogenous injection of FGF-2 in the zebrafish embryo, mimicking a pathological situation. As shown in this work, Solo F–OH diminished the angiogenic response of subintestinal vessels to exogenous FGF-2 in zebrafish, which was manifested by a decreased number of embryos that presented a strong or mild angiogenic response and an increased number of embryos that were unresponsive to FGF-2 stimulus. Taken together, our in vivo results suggest that Solo F–OH is capable of interfering not only with physiological angiogenesis, but also with the angiogenic response of the pre-existing vasculature toward an exogenous source of proangiogenic molecules, which could mimic a pathological activation of angiogenesis.

In response to proangiogenic molecules, quiescent endothelial cells transform into the so-called angiogenic phenotype, which is characterized by the activation of different cellular processes that culminate in the formation of the new vessel. The transduction of extracellular angiogenesis-activating signals is defined by a complex network of signaling pathways, which finally controls the response of the endothelial cell to proangiogenic stimulus. The pharmacological intervention on this signaling system constitutes a very interesting strategy in order to design new therapeutic approaches [29]. The PI3K/Akt pathway plays an essential role in the regulation of many of the processes related to the angiogenic phenotype in endothelial cells, such as proliferation, migration, differentiation, and morphogenesis [29,54,55]. Our data reveal that Solo F–OH prevents Akt phosphorylation in response to serum induction, therefore inhibiting the activation of the pathway. In addition, we show that Solo F–OH inhibits the phosphorylation of ERK1/2, which is the main proliferative pathway activated in endothelial cells in response to proangiogenic signals [29]. These results shed some light about the mechanism of action of Solo F–OH, pointing to the upstream components of these pathways as the major targets of the compound. Interestingly, both PI3K/Akt and ERK-MAPK pathways are implicated in the transduction of signals elicited by VEGF/VEGFR2 and FGF-2/FGFR, which are two master signaling systems that trigger the angiogenic response [29]. Our results suggest that Solo F–OH could inhibit angiogenesis by targeting these signaling systems, although additional studies are needed to clarify this point. 

In vitro studies about the possible interference of Solo F–OH with the VEGFR2 TK activity performed in our laboratory indicated that the maximal dose of the compound used in this work (20 μM) did only produce a modest and non-significant reduction of this activity, in contrast with the complete inhibition reached by Sunitinib 1 μM, which is a well-known characterized TK inhibitor [56]. This slight effect is not sufficient to explain the potent antiangiogenic activity in vivo reported in this work (50% of positive CAM at doses lower than 1 nmol/CAM). Nevertheless, these results reveal that Solo F–OH is not a direct inhibitor of VEGFR2 TK activity, without excluding the possibility of Solo F–OH to interfere with this receptor at other different levels. Additionally, in vivo ZFYM assay showed that Solo F–OH is able to inhibit the angiogenic response driven by FGF-2. Although not directly assessed in our work, these results suggest that Solo F–OH could interfere with the FGF-2/FGFR pathway, which is a well-known angiogenic signaling pathway. 

As a conclusion, in this work we evaluated for the first time a series of synthetic precursors of the solomonamides as candidates for antiangiogenic compounds, showing that Solo F–OH exhibits a potent antiangiogenic effect in vitro and in vivo. The activity of this compound to inhibit several key steps of angiogenesis suggests that the molecular structure of Solo F–OH could be an interesting starting point for the rational design and chemical synthesis of new molecules exhibiting more potent and specific antiangiogenic properties. Although additional studies are needed to investigate the exact molecular mechanism underlying the antiangiogenic activity of Solo F–OH, the interference of this compound with the activation of the ERK–MAPK and PI3K/Akt pathways indicates that this compound could target upstream components that are common to both signaling cascades. The results presented here suggest the potential therapeutic application of solomonamide derivatives and reinforce the value of marine products as drug candidates for the treatment of angiogenesis-related malignances.

## 4. Materials and Methods 

### 4.1. Materials

Solomonamide precursors were synthesized as described in Cheng-Sánchez et al. [17]. Cell culture media, penicillin/streptomycin, and amphotericin B were purchased from BioWhittaker (Walkersville, MD, USA) and fetal bovine serum (FBS) was purchased from BioWest (Kansas City, KS, USA). Plastics for cell culture were supplied by Thermo Scientific Nunc (ThermoFisher Scientific; Waltham, MA, USA). Matrigel was purchased from Corning (New York, NY, USA). Chemicals not listed in this section were obtained from Sigma-Aldrich (MERK) (Darmstadt, Germany). Fertilized chick eggs were purchased from Granja Santa Isabel (Córdoba, Spain). The zebrafish (*Danio rerio*) breeding colony (wild-type AB strain) was maintained at 28 °C as described in [57].

### 4.2. Cell Cultures

Bovine aortic endothelial cells (BAEC) were isolated as previously described [51] and maintained in Dulbecco’s modified Eagle’s medium (DMEM) containing glucose (1 g/L) and supplemented with 10% FBS (DMEM/10% FBS). Human fibrosarcoma cell line HT-1080 was obtained from the American Type Culture Collection (ATCC; Manassas, VA, USA) and maintained in Eagle’s Minimum Essential Medium (EMEM) supplemented with 10% FBS. Both culture media were supplemented with glutamine (2 mM), penicillin (50 IU/mL), streptomycin (0.05 mg/mL), and amphotericin B (1.25 mg/L). All the cell lines were maintained at 37 °C and humidified 5% CO2 atmosphere. 

### 4.3. Tubular-Like Structures Formation on Matrigel

Cellular suspensions of 5 × 10^4^ BAE cells in serum-free DMEM were added to a 96-well plate coated with 50 µL of Matrigel (10.5 mg/mL) in the presence of the indicated treatments for 5 h and photographed with a microscope camera Nikon DS-Ri2 coupled to a Nikon Eclipse Ti microscope (Nikon, Tokyo, Japan). Each concentration was tested in duplicate, and staurosporine 2 µM was used as a routine positive assay control [58]. For the disruption assay, tubular-like structures were formed following the same protocol for the control conditions; then, the indicated concentrations of Solo F–OH were added. After a further incubation time of 90 min, cultures were observed and photographed. Combretastatin-4-phosphate (CA4P) was used as positive control of the disruptor antiangiogenic drug [59]. 

### 4.4. Wound Healing Assay

Confluent BAEC monolayers in six-well plates were wounded with pipet tips with two perpendicular diameters, giving rise to two acellular 1 mm-wide lanes per well. Then, complete medium in the absence (controls) or presence of different concentrations of Solo F–OH was added. Wounded areas were observed and photographed after 0 h, 4 h, and 7 h of incubation with a microscope camera Nikon DS-Ri2 coupled to a Nikon Eclipse Ti microscope (Nikon, Tokyo, Japan). The migration of BAEC into the cell-free area was quantified by Image J software and represented as the percentage of wounded area in the correspondent time normalized to the initial wounded area (time 0) for each experimental condition. 

### 4.5. Cell Invasion Assay

The invasion of endothelial cells was assayed by using 8.0-μm pore size transwell inserts coated with 100 µL of Matrigel 0.12 mg/mL solution. 10^5^ BAE cells, in the absence or presence of the indicated concentrations of Solo F–OH, were added to the upper chamber of the transwells in the absence of serum, and the lower chamber was filled with 20% FBS DMEM. After 16 h of incubation, invading cells were fixed in 4% paraformaldehyde and stained with a 1% crystal violet solution in 2% ethanol. Cells were photographed with a microscope camera Nikon DS-Ri2 coupled to a Nikon Eclipse Ti microscope (Nikon, Tokyo, Japan).

### 4.6. Zymographic Assays for MMP-2 and MMP-9 Detection

Zymographies for matrix metalloproteinases MMP-2 and MMP-9 activities were performed in both conditioned media and cellular extracts of endothelial (BAEC) and tumor (HT1080) cell lines as described in Fajardo et al. [60]. Briefly, cells seeded in six-well plates were incubated in serum-free culture medium with 0.1% BSA containing 200 KIU of aprotinin/mL and the correspondent treatment. After 24 h of incubation, conditioned media were collected, and cell lysates were obtained. Duplicates were used to determine the cell number, and samples were normalized for equal loading. In order to detect the gelatinolytic activity of MMP-9 and MMP-2, samples were loaded in non-reducing SDS/PAGE gels containing gelatin (1 mg/mL). After electrophoresis, gels were incubated overnight at 37 °C in a substrate buffer (50 mM of Tris/HCl, pH 7.4, supplemented with 1% Triton X-100, 5 mM CaCl_2_, and 0.02% Na_3_N) and stained with Coomassie blue R-250. The bands of gelatinase activity could be detected as non-stained bands in a dark, stained background. The size and intensity of the bands were quantified using Image J software.

A variant of this method was used to obtain complementary information about the direct inhibition of the tested compound on MMP-2 gelatinase activity: samples of conditioned media of untreated BAEC were subjected to gelatin zymography and, after electrophoresis, 20 μM of Solo F–OH was added to the substrate buffer. Detection of the degrading bands and quantification were performed as described above.

### 4.7. Chick Chorioallantoic Membrane (CAM) Assay 

Fertilized chick eggs were incubated horizontally at 38 °C in a humidified incubator, windowed by day 3 of incubation, and processed by day 8. Solo F–OH was added to a 1.2% solution of methylcellulose in water, and 10-μL drops were dried on a Teflon-coated surface under a laminar flow hood. Then, methylcellulose discs were implanted onto the CAM, and the eggs were sealed with adhesive tape and returned to the incubator for 48 h. Negative controls were always made with DMSO mixed with the methylcellulose, and aeroplysinin-1 (3 nmol/CAM) was used as a routine positive control of antiangiogenic compound [47]. After the incubation, the CAM was examined under a stereomicroscope and photographed with a Nikon DS-Ri2 camera. The results were analyzed by two different observers, and the assay was scored positive when both of them reported a significant reduction of vessels in the treated area. 

### 4.8. FGF-2 Induced Angiogenesis Zebrafish Yolk Membrane (ZFYM) Assay

For the FGF-2 induced angiogenesis zebrafish yolk membrane (ZFYM) [61], 24 hpf embryos were exposed to 1-phenyl-2-thiourea (PTU) to prevent the pigmentation. At 48 hpf, embryos were manually dechorionated with forceps, anesthetized with tricaine (0.016%), and injected into the perivitelline space with 2 mL FGF-2 (1 mg/mL). The injection was performed in the proximity of developing subintestinal vessels (SIVs) using borosilicate needles and a Picospritzer microinjector (Eppendorf, Hamburg, Germany). After injection, embryos were incubated for 24 h more in the absence or the presence of Solo F–OH. Finally, embryos were fixed in 4% paraformaldehyde (PFA), stained for endogenous alkaline phosphatase (AP) activity, and photographed under a Leica MZ16 F stereomicroscope equipped with a DFC480 digital camera and ICM50 software (Leica, Wetzlar, Germany). Evaluation of the angiogenic response was performed by assigning negative (–, no response to FGF-2 injection), positive (+, mild response), or very positive (++, strong response) scores to the embryos.

### 4.9. Western Blot Analysis 

BAE cells were starved in serum-free for 16 h. After one-hour treatment with Solo F-OH at 10 and 20 μM in the same conditions, cells were induced with 10% of FBS for 10 min. Protein lysates were obtained in radioimmunoprecipitation assay buffer (RIPA) (50 mM of Tris, pH 7.4, 150 mM of NaCl, 1% Triton X-100, 0.25% sodium deoxycholate, 1 mM of EDTA) containing phosphatase activity inhibitors (30 mM of sodium fluoride, 1 mM of sodium orthovanadate, 30 mM of β-glycerophosphate) and protease activity inhibitors (Complete mini, Roche, Mannheim, Germany). Protein concentration was determined using the Lowry method. Samples were subjected to SDS-PAGE electrophoresis and transferred to nitrocellulose membranes. After blocking in TBS-T containing 10% non-fatty dry milk, membranes were hybridized with primary antibodies overnight at 4 °C (cell signaling; rabbit anti-Phospho-Akt (Ser473) #9271; rabbit anti-Akt #9272; rabbit anti-Phospho-p44/42 MAPK (ERK1/2) (Thr202/Tyr204) (clone D13.14.4E) #4370; rabbit anti-p44/42 MAPK (ERK1/2) (137F5) #4695; mouse anti-α-Tubulin (DM1A) #3873). Following one hour of incubation with horseradish peroxidase-conjugated secondary antibodies (MERK; goat anti-rabbit IgG HRP Linked Whole Antibody #NA934V; goat anti-mouse IgG (whole molecule)–Peroxidase antibody #A4416) at room temperature, immunoreactive bands were detected with SuperSignal West Pico Chemiluminescent Substrate (Pierce, Rockford, USA) and quantified by ImageJ software. The phosphorylated/total protein ratios were expressed as the percentage of the ratio in serum-stimulated samples in the absence of Solo F–OH. 

### 4.10. In Vitro Measure of VEGFR2 TK Activity

VEGFR2 TK activity was measured in vitro using the VEGFR2 (KDR) Kinase Assay Kit (BPS Bioscience, San Diego, CA, USA). Solo F–OH was tested at 20 μM according to the manufacturer’s instructions, determining the percentage of remaining TK activity after 45 min of incubation at 30 °C. Sunitinib 1 μM was used as positive control of inhibition in the same experimental conditions. 

### 4.11. Ethical Statement

The animal procedures considered in this project were performed in strict compliance with the European Communities Council Directive 2010/63/EU regulating the use and care of laboratory animals. Experimental procedures with chick embryos were performed at the University of Málaga (Spain) and were conducted in accordance with the Spanish Legislation in compliance with European Community regulation. The protocols were approved by the Ethics Committee for Animal Experiments of the University of Málaga. Zebrafish (*Danio rerio*) breeding colony (wild-type AB strain) was maintained at the Zebrafish Facilities of the University of Brescia (Italy). Experimental procedures with zebrafish embryos were performed at the University of Brescia and were conducted in accordance with the Italian legislation for the animal experimentation. Efforts were made to reduce the number of animals used and minimize animal suffering. Furthermore, animals were anesthetized when it was likely they could be subjected to pain, and they were killed by a method that ensured the least effect on their welfare. 

### 4.12. Statistical Analysis

Results are expressed as the mean ± SD of three independent experiments. Data sets were checked to follow a normal distribution, and statistical significance was determined using the two-sided unpaired Student *t*-test (SPSS software). Values of *p* < 0.05 were considered to be statistically significant. Significance was indicated as follows: ****p < 0.0001, ***p < 0.001, **p < 0.01, *p < 0.05.

## Figures and Tables

**Table 1:** Summary of the results obtained in the primary in vitro screening assays for the complete series of synthetic solomonamide precursors. BAEC: bovine aortic endothelial cells, MIC: minimum inhibitory concentration.

Compound	IC_50_^1^ (μM) in BAEC [17]	MIC Tubular Like-Structures Formation (μM)	MIC Wound-Healing Assay (μM)
Solo-1	>100	>50	>50
Solo-2	43.8 ± 1.2	>50	>50
Solo-3	>100	>50	>50
Solo-4	69.6 ± 12.5	50	>50
Solo-5	>100	>50	>50
Solo-6	>100	>50	>50
Solo F–OH	18.1 ± 2.2	1	10
Solo F–Bz	>100	>50	>50

^1^ IC_50_ values were determined after 72 h of treatment with Solo F–OH in proliferating conditions.

**Table 2:** Inhibition of in vivo angiogenesis in the CAM assay by Solo F–OH. The table summarizes the evaluation of the effect of different doses of the compound in the CAM of chicken embryos. The CAM was scored positive when angiogenesis inhibition was observed.

CAM Assay		
Solo F–OH (nmol/CAM)	Positive/Total	% Inhibition
0	0/11	0
0.1	1/7	14
0.5	2/7	29
1	6/9	67
5	9/12	75
10	10/10	100

**Table 3:** Inhibition of in vivo angiogenesis in the zebrafish yolk membrane (ZFYM) assay by Solo F–OH. The table summarizes the observed effect of different doses of the compound in FGF-2-induced angiogenesis on the subintestinal vessels (SIVs) of zebrafish embryos. Embryos were scored as – (no response to FGF-2), + (mild response) or ++ (strong response).

ZFYM Assay				
FGF-2 Induction	Solo F–OH (µM)	Score (%)		
		– / Total(%)	+ / Total(%)	++ / Total(%)
None	0	20/20 (100)	0/20 (0)	0/20 (0)
2 ng	0	5/21 (23.8)	10/21 (47.6)	6/21 (28.6)
2 ng	5	9/19 (47.4)	7/19 (36.8)	3/19 (15.8)
2 ng	10	13/23 (56.5)	9/23 (39.1)	1/23 (4.3)
